# Membrane Cholesterol Content and Lipid Organization Influence Melittin and Pneumolysin Pore-Forming Activity

**DOI:** 10.3390/toxins14050346

**Published:** 2022-05-16

**Authors:** Shamish Ganpule, Akshay Kumar Vijaya, Aleksandra Sukova, Giulio Preta

**Affiliations:** Institute of Biochemistry, Life Science Center, Vilnius University, LT-10257 Vilnius, Lithuania; shamish.ganpule@gmc.vu.lt (S.G.); akshay.vijaya@gmc.vu.lt (A.K.V.); sukova.aleksandra@gmail.com (A.S.)

**Keywords:** melittin, cholesterol-dependent cytolysins, cholesterol amphiphilic molecules, statins, lipid rafts

## Abstract

Melittin, the main toxic component in the venom of the European honeybee, interacts with natural and artificial membranes due to its amphiphilic properties. Rather than interacting with a specific receptor, melittin interacts with the lipid components, disrupting the lipid bilayer and inducing ion leakage and osmotic shock. This mechanism of action is shared with pneumolysin and other members of the cholesterol-dependent cytolysin family. In this manuscript, we investigated the inverse correlation for cholesterol dependency of these two toxins. While pneumolysin-induced damage is reduced by pretreatment with the cholesterol-depleting agent methyl-β-cyclodextrin, the toxicity of melittin, after cholesterol depletion, increased. A similar response was also observed after a short incubation with lipophilic simvastatin, which alters membrane lipid organization and structure, clustering lipid rafts. Therefore, changes in toxin sensitivity can be achieved in cells by depleting cholesterol or changing the lipid bilayer organization.

## 1. Introduction

The cellular plasma membrane is a complex organization of different lipids and proteins involved in the regulation of essential cell activities including signal transduction, trafficking and apoptosis [1,2]. Cell membranes also act as a portal for the entry of a multitude of pathogens ranging from viruses to intracellular bacteria with membrane cholesterol representing a key component, capable of influencing the host–pathogen interaction [3,4,5]. A hallmark of cholesterol organization in biological membrane is its ability to self-organize, together with sphingolipids, in specific microdomains known as lipid rafts, concentrating platforms for membrane receptors but also preferred entry doors for virus, bacteria and pore-forming toxins [6,7,8]. For example, SARS-COV-2 was shown to engage with different receptors enriched in lipid rafts [9,10].

**Figure 1:**
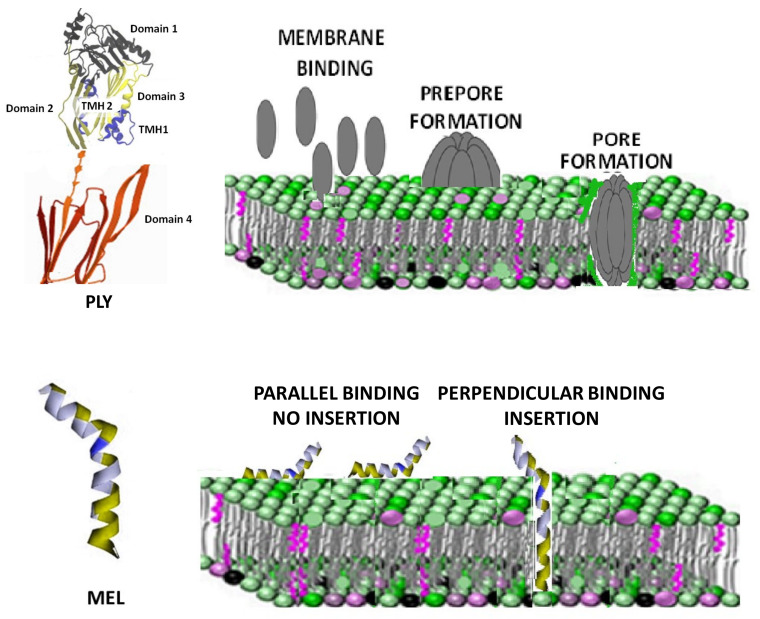
Structural organization and mechanism of pore formation by MEL and PLY. PLY, a member of the CDC family, has four functional domains and its pore formation mechanism is a multistep process which includes binding of the monomeric forms, oligomerization and insertion into the membrane of incomplete and complete pores. PLY-domain pictures adapted from Marshall JE et al. [29]. The image is licensed under a Creative Commons Attribution 4.0 International License. Link: Creative Commons — Attribution 4.0 International — CC BY 4.0 (accessed on 26 January 2022).MEL is a small 26-residue amphipathic peptide that can bind the membrane either in a parallel orientation (inactive form) or in the perpendicular orientation (active form). The perpendicular orientation causes membrane insertion and pore formation.

Cholesterol-dependent cytolysins (CDCs) are a common virulence factor secreted by Gram-positive bacteria, with a high affinity for the cholesterol of cell membranes where they form 30–50 nm diameter pores [11,12,13,14]. Pneumolysin (PLY) from *Streptococcus pneumoniae* is the classic representative of this family and recent findings revealed lipid raft area as the preferred site for its cholesterol binding [15]. *PLY* has four functional domains: domains 1 and 3 are linked via domain 2 to the membrane-sensing C-terminal domain 4 containing the highly conserved undecapeptide sequence involved in membrane-bound cholesterol recognition and binding [16,17]. PLY monomers bind to the targeted cell membranes and interact with other PLY molecules, packing side-by-side to form the prepore complex. After undergoing further conformational changes, the final ring pore of several PLY subunits inserts into the membrane (Figure 1, upper panel) [18,19]. Cholesterol depletion by extraction using methyl-β-cyclodextrin (mβCD) or statin-mediated inhibition of cholesterol synthesis are two recognized strategies to decrease cell sensitivity to the action of CDCs [20,21]. On the other hand, membrane cholesterol depletion was observed to increase the damage induced by melittin (MEL), a lytic 26-amino acid peptide, which is the main toxic component in the venom of the honey bee *Apis mellifera* [22]. Rather than binding to a specific receptor, the peptide MEL interacts with the lipids in the membrane, disrupting the lipid bilayer in a concentration-dependent way [23]. Several models of MEL-dependent pore formation were proposed during the years using both biological and model membranes. Currently, it is believed that MEL can bind the membrane either in parallel orientation (inactive form) or in the perpendicular orientation, which is capable of inducing pores (Figure 1, lower panel) [24,25]. Membrane cholesterol influences the structure and dynamics of lipid membranes, altering fluidity, rigidity and thickness [26]. Changes in these membrane properties are also able to influence MEL interaction with the membrane and its ability to penetrate the lipid bilayer [27,28]. The purpose of this manuscript was to investigate the activity of PLY and MEL after treatment with compounds depleting cholesterol (mβCD) or changing its membrane organization (simvastatin). Using red blood cells and a continuous tumor cell line, we were able to demonstrate that the two toxins showed a completely different response to changes in the membrane cholesterol content, with PLY showing a decreased activity and MEL an increased one. Moreover, cell sensitivity to toxins is also influenced by changes in the lipid bilayer organization since treatment with lipophilic simvastatin, inducing lipid raft reorganization, modifies the pore-forming activities of PLY and MEL. Further immunofluorescence analysis revealed that simvastatin-mediated raft clustering is connected with a decrease in the levels of PLY binding to the cell membrane.

## 2. Material and Methods

### 2.1. Hemolysis Assay

Hemolysis assay was performed as a functional measure of cytolytic activity, using previously reported methods [30,31]. To prepare the washed red blood cells, commercially available defibrinated horse blood (Thermo Fisher Scientific, Waltham, MA, USA) was centrifuged at 1500× *g* for 10 min, 4 °C. The supernatant was discarded, and the pelleted red blood cells were resuspended in PBS. A sample of 125 μL of the red blood cell pellet was added to 50 mL PBS to make a 0.25% final solution The samples were incubated at 37 °C for 1 h with MEL and PLY before centrifuging at 1500× *g* for 5 min at room temperature to pellet intact red blood cells. Finally, 200 μL of the resulting supernatant was transferred into a flat-bottomed 96-well plate and the OD_405_ of the supernatant was measured in a plate reader. In the experiments with mβCD, RBCs were pretreated for 1 h with different concentrations of mβCD (0.4, 0.5, 0.6 mM) before addition of the toxins.

### 2.2. Cell Culture

HepG2 hepatocarcinoma cell line was used in this study. Cells were maintained in DMEM + GlutaMAX medium supplemented with 10% fetal bovine serum (FBS), 100 IU/mL penicillin and 100 μg/mL streptomycin (all from Thermo Fisher Scientific) in a humified incubator at 37 °C with 5% CO_2_. Before experiments, cells were seeded in 12 or 24-well plates in complete medium to favor cell attachment and subsequently complete medium was replaced with serum-free medium, since FBS used in the present study contained high levels of cholesterol, which may alter the results by binding to pneumolysin. 

### 2.3. MTT Assay

Cell survival was monitored by the mitochondria-dependent reduction of 3-(4,5-dimethylthiazol-2-yl)-2,5-diphenyltetrazolium bromide (MTT) to formazan, as described previously [12,32]. Briefly, for the MTT assay (Thermo Fisher Scientific), once the supernatants were removed, 0,2 mg/mL MTT in DMEM was added and incubated with the cells for 1 h; the MTT was then removed, and cells were lysed using DMSO for the measurement of OD at 570 nm using Infinite M200 PRO (Tecan, Mannedorf, Switzerland) spectrometer. Reactions were performed at different concentrations of PLY and MEL and different time points as reported in results section. Data are expressed as percentage of cell survival compared to 100% of control cells.

#### 2.3.1. Lactate Dehydrogenase

Lactate dehydrogenase was quantified using CyQuant LDH Cytotoxicity assay (Thermo Fisher Scientific). LDH released by damaged cells is quantified by a coupled enzymatic reaction in which LDH catalyzes the conversion of lactate to pyruvate via reduction of β-nicotinamide adenine dinucleotide sodium salt (NAD+) to NADH, which is detected by NADH-dependent reduction of a tetrazolium salt to formazan. The level of formazan is directly proportional to the amount of LDH released in the extracellular milieu and can be detected by reading optical density at. Results are presented as fold increase in LDH level compared to control cells and toxin-treated and maximal LDH activity were calculated according to manufacture instructions.

#### 2.3.2. Anti-PLY Monoclonal Antibodies

Anti-PLY monoclonal antibodies were kindly provided by Dr. Kučinskaitė-Kodzė.

mAbs were developed according to standard procedure including immunization of mice, cell hybridization, hybridoma selection, cloning and evaluation of mAb specificity to PLY, as previously described [33]. The clone 3F3 was selected for immunofluorescence and Western blotting studies.

#### 2.3.3. Immunofluorescence

To analyze nuclear morphological changes after toxin treatments, HepG2 cells were cultured overnight in 8-well chamber slides (Thermo Fisher Scientific) in complete medium. The following day, complete medium was replaced with serum-free medium and cells were pretreated for 1 h with 1 mM mβCD before the addition of PLY (3 h) or MEL (18 h). After treatment, cells were washed with PBS and fixed in 4% paraformaldehyde for 30 min. The slides then were rehydrated in PBS for 30 min, permeabilized and blocked with a solution containing 0.1% triton and 1% bovine serum albumin (BSA). The slides were washed and stained with Gold Antifade Mountant with DAPI (Thermo Fisher Scientific) and analyzed with Zeiss Axio Observer Z1 (Carl Zeiss, Germany): at least 100 cells per treatment, were scored for the presence of nuclei with apoptotic/necrotic phenotype. For lipid raft staining, HepG2 cells were cultured overnight in 8-well chamber slides before treatment with simvastatin or pravastatin. After 1 h incubation, slides were treated with anti-cholera toxin B, FITC conjugated (30’ at 4 °C) and cross-linked with anti-CTB antibody (30’ at 4 °C) (Sigma Aldrich, St. Louis, MO, USA). Cell were fixed with 4% PFA, permeabilized with PBS containing 0.1% Triton X-100, mounted with DAPI. Picture analysis was performed using Image J software version 1.53 (National Institutes of Health, Bethesda, MD, USA) and lipid raft clustering was evaluated counting on the green channel, the total number of spots. For PLY-binding studies, HepG2 cells were cultured overnight in 8-well chamber slides before treatment with simvastatin or mβCD. After 1 h incubation, slides were treated with PLY (50 ng/mL) for 30 min before addition of an anti-PLY mouse monoclonal antibody followed by an anti-mouse secondary antibody Alexa 594 conjugated. Slides were visualized using a LEICA TCS SP8 STED microscope (Leica Microsystems, Wetzlar, Germany). 

### 2.4. Western Blotting

HepG2 cells were cultured overnight in complete medium before treatment with simvastatin or pravastatin in serum-free media. After 1 h incubation, cells were treated with PLY (50 ng/mL) for 30 min before lysis with urea buffer. For protein detection, Western blotting was performed according to standard procedures. The following primary antibodies were used: rabbit anti-mouse alpha Tubulin (Abcam, Cambridge, UK) and mouse anti-Pneumolysin (clone 3F3). After incubation with the appropriate secondary antibodies, the membranes were incubated with Pierce enhanced chemiluminescence substrate for the detection of HRP (Thermo Fisher Scientific). Quantification of the bands was done using the ImageJ software.

### 2.5. Toxins and Reagents

Recombinant pneumolysin (PLY) was kindly provided by Dr. M. Plečkaitytė (Vilnius University, Vilnius, Lithuania) and was generated as previously described [34]. The statins simvastatin and pravastatin as well as methyl-beta cyclodextrin and melittin were purchased from Sigma Aldrich.

### 2.6. Cholesterol Measurement

Cellular cholesterol content was measured in RBCs and HepG2 cells using the Amplex Red Cholesterol Assay Kit (Invitrogen, Waltham, MA, USA), according to the manufacturer’s instructions. RBCs were treated for 1 h with three different concentrations of mβCD (0.4, 0.5 or 0.6 mM) while HepG2 cells were treated for 1 h with 1 mM mβCD. Data are presented as % of decrease in total cholesterol levels compared to control.

### 2.7. Statistical Analysis

MTT, LDH and hemolysis data are presented as the arithmetic mean (SD) of at least 3 independent experiments. Statistical analysis was performed using GraphPad Prism, version 9 (GraphPad Software Inc., La Jolla, CA, USA), and data were analyzed using one-way ANOVA Sidak’s multiple comparisons test unless otherwise specified. Significance was ascribed at *p* < 0.05. 

## 3. Results

### 3.1. Cholesterol Depletion Has Inverse Effects in MEL and PLY-Treated RBC

**Figure 2:**
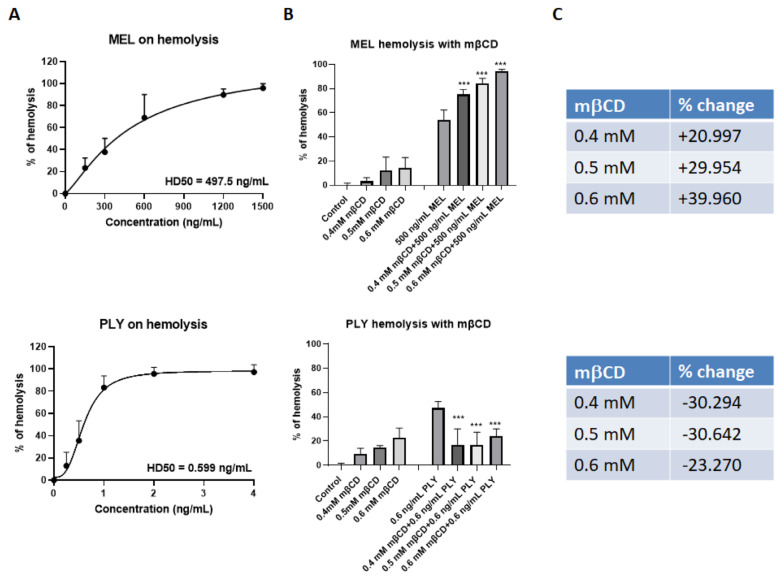
Cholesterol depletion has opposite effects in MEL and PLY-treated RBC. (**A**) The hemolytic activity of the recombinant MEL (upper panel) and PLY (lower panel), were tested at different concentrations, using defibrinated horse red blood cells. Briefly, RBCs were centrifuged at 1500× *g* for 10 min, 4 °C. The supernatant was discarded, and the pelleted RBCs were resuspended in PBS. A sample of 125 μL of the red blood cell pellet was added to 50 mL PBS to make a 0.25% final solution The samples were incubated at 37 °C for 1 h with MEL and PLY before centrifuging at 1500× *g* for 5 min at room temperature to pellet intact red blood cells. Finally, 200 μL of the resulting supernatant was transferred into a flat-bottomed 96-well plate and the OD_405_ of the supernatant was measured in a plate reader and HD50 was calculated based on 5 experiments. (**B**) RBCs prepared as in 2A were pretreated for 1 h with different concentrations of mβCD (0.4 mM, 0.5 mM, 0.6 mM) before adding 500 ng/mL MEL (upper panel) or 0.6 ng/mL PLY (lower panel). Data are presented as the arithmetic mean (SD) and statistical analysis was performed using one-way ANOVA Sidak’s multiple comparisons test (*** *p* < 0.001). (**C**) Changes in % of lysis induced by pre-incubation with different concentrations of mβCD.

We tested the hemolytic activity of the recombinant PLY and of MEL, at different concentrations, using defibrinated horse red blood cells. Both toxins cause a dose-dependent increase in % of hemolysis, but the concentration required to induce 50% RBC lysis (HD50) was much lower for PLY compared to MEL (Figure 2A). This difference is mainly related to the high amount of cholesterol in membrane erythrocytes (∼45 mol%) which inhibits the lytic activity of MEL, as previously demonstrated by studies using both biological and artificial model membranes [22,27,28]. The use of mβCD to deplete cholesterol from the membrane of erythrocytes is largely documented in the literature [22,35,36].

Indeed, RBC treatment for 1 h with three different concentrations of mβCD (0.4 mM, 0.5 mM, 0.6 mM) was able to deplete cholesterol from the RBC membranes in a dose-dependent way (Supplementary Figure S1B, left panel). Membrane cholesterol depletion by mβCD increased the lytic properties of MEL up to 40% (Figure 2B,C, upper panel), and protected against PLY-induced hemolysis (Figure 2B,C, lower panel) as previously reported for several components of CDCs, both in RBCs and cell lines derived from different tissue [20,31,37,38]. These experiments confirmed that the pore-forming activity for these two toxins is inversely influenced by membrane cholesterol content.

### 3.2. Cholesterol Depletion Has Inverse Effects on Cell Survival of PLY- and Mellitin-Treated HepG2

**Figure 3:**
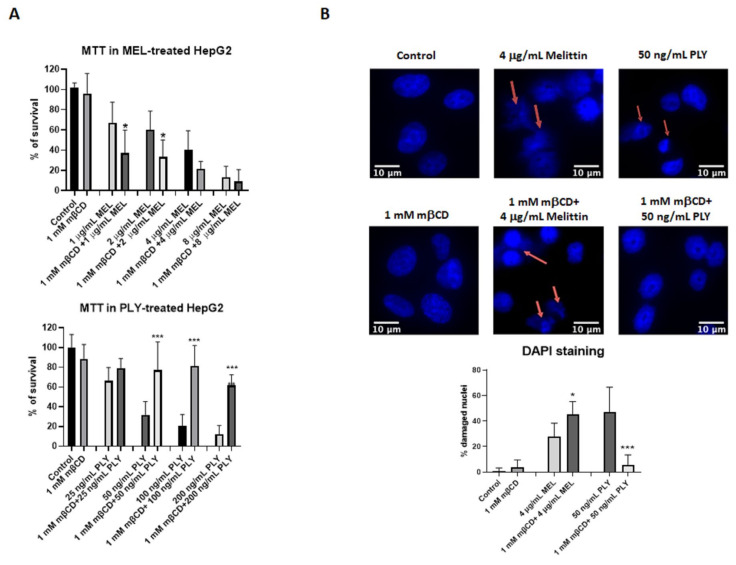
Cholesterol depletion has opposite effects on cell survival of HepG2 treated with MEL or PLY. (**A**) Cell survival was monitored in HepG2 cells by MTT. Briefly, cells were pretreated with 1 mM mβCD, before addition of different concentrations of MEL (upper panel) or PLY (lower panel). DMSO was used to solubilize the cells for the measurement of OD at 570 nm using a spectrometer. Data are presented as the arithmetic mean (SD) and statistical analysis was performed using one-way ANOVA Sidak’s multiple comparisons test (* *p* < 0.05, *** *p* < 0.001). (**B**) HepG2 cells were cultured overnight in 8-well chamber slides in complete medium. The following day, complete medium was replaced with serum-free medium and cells were pretreated with mβCD before the addition of PLY (3 h) or MEL (18 h). After treatment, cells were washed with PBS and fixed in 4% paraformaldehyde for 30 min. The slides were rehydrated in PBS, permeabilized and blocked with a solution containing, 0.1% triton and 1% bovine serum albumin (BSA) before addition of DAPI. A Zeiss Axio Observer Z1 was used to analyze nuclear morphological change: at least 100 cells per treatment were scored for the presence of nuclear changes such as nuclear fragmented bodies, condensed or deformed nuclei (red arrows). Data are presented as the arithmetic mean (SD) and statistical analysis was performed using one-way ANOVA Sidak’s multiple comparisons test (* *p* < 0.05, *** *p* < 0.001). Scale bar 10 μm.

To further analyze how the activities of the two toxins change according to membrane cholesterol content, we performed cell survival assay (MTT) using HepG2, a human hepatoma cells with high proliferation rates and an epithelial-like morphology, commonly used in cytotoxicity studies with pore-forming toxins [39,40]. First of all, we performed a time kinetic using two different concentrations of MEL and PLY to understand how their activity influences cell viability. Previous studies showed that melittin, already at concentrations as low as a few nanomoles per liter, induces transient pores that allow the leakage of atomic ions [41,42]. However, with MEL, the time required to observe significant changes in cell survival are higher compared with PLY (18 h vs. 3 h, Supplementary Figure S1A), in line with other inhibitory studies on cancer cell lines [43,44]. We then performed experiments where we pre-incubated for 1 h HepG2 cells with 1 mM of the cholesterol-extracting agent mβCD before adding MEL or PLY. This concentration of mβCD was able to significantly deplete the membrane cholesterol content of HepG2 according to the Amplex Red Cholesterol assay kit measurements (Supplementary Figure S1B, right panel). As shown in Figure 3A, mβCD-mediated membrane-cholesterol depletion results in a decreased (MEL) or increased (PLY) cell survival, confirming the diametrically opposing role of cholesterol for the pore-forming ability of these two toxins. Immuno-cytochemistry with DAPI staining was then performed to analyze cells with signs of damaged nuclei (DNA fragmentation and nuclear condensation). Pretreatment with mβCD has a protective effect in PLY-treated HepG2 cells with the % of damaged nuclei comparable with control cells, while for MEL, the pretreatment resulted in an increase of damaged nuclei (Figure 3B). Similar results were observed using a cell survival assay to quantify the release of LDH, occurring after pore formation. LDH levels were increased in MEL-treated cells pre-incubated with mβCD, while total protection occurred in PLY-treated cells with LDH levels similar to control ones (Supplementary Figure S1C).

### 3.3. Statins Influence Survival in PLY and MEL-Treated HepG2

**Figure 4:**
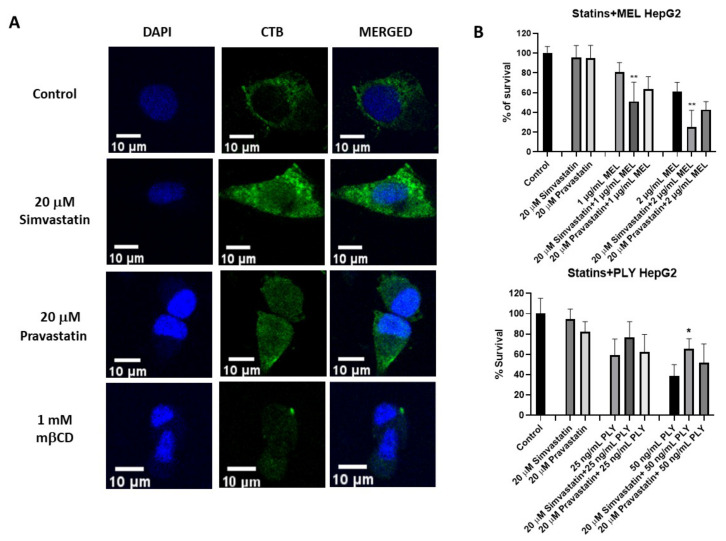
Simvastatin-induced raft clustering influence survival in PLY and melittin-treated HepG2. (**A**) To stain lipid rafts, HepG2 cells were cultured overnight in 8-well chamber slides before treatment with 20 μM simvastatin, 20 μM pravastatin or 1 mM mβCD. After 1 h incubation, slides were treated with anti-FITC CTB conjugate at 4 °C and cross-linked with anti-CT-B antibody conjugate also at 4 °C. Cell were fixed with 4% PFA, permeabilized with PBS containing 0.1% Triton X-100, mounted with DAPI and visualized using a LEICA TCS SP8 STED microscope. Scale bar 10 μm. (**B**) To evaluate the effect of statin treatment on the survival of MEL and PLY-treated cells, we cultured HepG2 overnight in 8-well chamber slides. The day after, complete medium was replaced by serum-free medium, before treatment with 20 μM simvastatin, 20 μM pravastatin for 1 h followed by different concentrations of MEL (upper panel) or PLY (lower panel). Cell survival was monitored by MTT and data are expressed as percentage of cell survival compared to 100% of control cells. Data are presented as the arithmetic mean (SD) and statistical analysis was performed using one-way ANOVA Sidak’s multiple comparisons test (* *p* < 0.05, ** *p* < 0.01).

Statins are selective inhibitors of cholesterol biosynthesis, used worldwide for cholesterol lowering in the primary and secondary prevention of cardiovascular diseases. However, increasing evidence has highlighted additional effects of statins which are independent of cholesterol lowering (i.e., pleiotropic effects) [45,46]. For example, recent data showed statins’ ability to influence membrane lipid organization via clustering of lipid rafts [31]. This lead to the interesting possibility that not only cholesterol depletion but also cholesterol remodeling could influence cell sensitivity to pore-forming toxins. To analyze if changes in membrane lipid structure are able to influence PLY and MEL activity on HepG2, we selected simvastatin and pravastatin, two statins with different lipophilicity. Simvastatin is highly lipophilic with log P 4,46, while pravastatin is hydrophilic with log P 1,65. Log P, the octanol/water partition coefficient, is the most commonly used measure for the lipophilicity of a compound [47,48]. We initially performed lipid raft staining using the specific marker cholera toxin B (CTB) to confirm by confocal analysis that simvastatin was able to enhance clustering of lipid rafts in larger microdomains compared to untreated or pravastatin-treated cells, as observed previously in a different cell line (Figure 4A and Figure S2A) [31]. Simvastatin-induced raft clustering resulted in a decreased number of particles compared to control or pravastatin-treated cells as evaluated by Image J quantification (Supplementary Figure S2B). 

**Figure 5:**
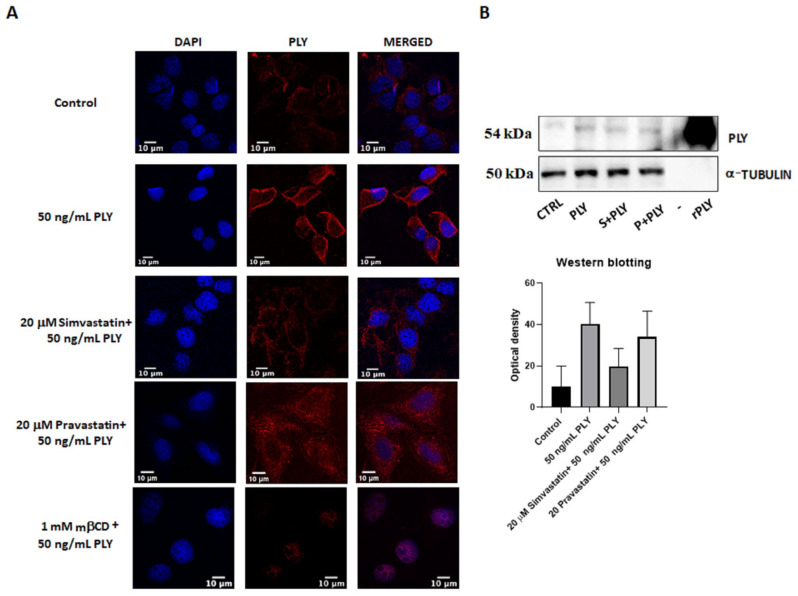
Simvastatin-induced raft clustering reduces PLY binding to the membrane. (**A**) To stain PLY, HepG2 cells were cultured overnight in 8-well chamber slides before treatment in serum-free medium with 20 μM simvastatin, 20 μM pravastatin or 1 mM mβCD. After 1 h incubation, cells were treated for 30 min with 50 ng/mL PLY before addition of the antibody 3F3 anti-PLY and of an anti-mouse secondary antibody Alexa 594 conjugated. Cells were fixed with 4% PFA, permeabilized with PBS containing 0.1% Triton ® X-100, mounted with DAPI and visualized using a LEICA TCS SP8 STED microscope. Scale bar 10 μm. (**B**) Western blotting analysis of HepG2 treated with 20 μM simvastatin or 20 μM pravastatin before addition of PLY for 30 min. Lysates were subjected to SDS electrophoresis, incubated against specific primary antibody anti-PLY and a-tubulin (loading control) and membranes were developed using the enhanced chemiluminescence substrate. Quantification of the bands, based on three independent experiments, were performed using ImageJ.

Next, we performed MTT assay where pretreatment of HepG2 for 1 h with simvastatin and pravastatin was followed by addition of PLY and MEL, using the previously established time points. Incubation with 20 μM simvastatin or pravastatin alone had no effect on cell viability of HepG2 (Figure 4B). However, the lipophilic simvastatin was able to influence PLY and MEL activity, causing a decrease in cell survival for MEL (Figure 4B, upper panel) and a slight increase for PLY (Figure 4B, lower panel). Pre-incubation with hydrophilic pravastatin did not induce significant changes for either toxin. To further investigate how changes in lipid organization affect cell sensitivity to PLY, we performed immunofluorescence analysis in HepG2, using previously characterized antibodies anti-PLY [33]. Cells were pretreated with simvastatin or mβCD for 1 h, before addition of the toxin. Immunofluorescence analysis revealed that the intensity of the staining was reduced in simvastatin or mβCD pretreated cells, compared to cells treated with PLY alone (Figure 5 and Figure S2C). To confirm the role of simvastatin in decreasing PLY-binding to the membranes of target cells, HepG2 cells were pretreated with simvastatin or pravastatin for 1 h before addition of PLY and cell lysates were subjected to Western blotting analysis. As shown in Figure 5B the total amount of PLY detected in cells pretreated with simvastatin was lower compared to the ones pretreated with pravastatin or with the toxin alone. Therefore, simvastatin-induced raft clustering decreases the overall amount of PLY binding to the membrane and influences the following steps of pore formation. These data confirmed that not only a change in cholesterol content but also changes in membrane raft organization are capable of influencing toxin pore-forming ability and, consequently, the viability of cells.

## 4. Discussion

In the current manuscript, we provide evidence that both changes in cholesterol content and lipid organization are capable of influencing cellular responses to PLY, a classic representative of the CDC family [19], and MEL, a honeybee venom-derived antimicrobial peptide [49]. Cholesterol affects the activity of microbial toxins in a direct, specific way, or it may exert indirect effects because of its ability to influence membrane fluidity and raft organization in the cytoplasmic membrane [50,51]. Pore formation on the cell membrane surface is a multistep process involving changes in lipid orientation, distribution, or fluidity, as well as variations in lipid phase organization [52,53]. Therefore, any factor capable of influencing these membrane properties could affect membrane pore-forming activity. Beyond their cholesterol lowering effect, statins have received increased attention due to their ability to influence membrane organization. Many of these properties were revealed using artificial systems including supported lipid bilayers, large unilamellar vesicles and tethered bilayer lipid membranes [31,54,55,56,57,58]. In the current manuscript, using PLY and MEL, we provide evidence that changes in cell sensitivity to these two toxins could be achieved not only via cholesterol-depletion (using mβCD) but also via membrane lipid reorganization (using lipophilic simvastatin). Specifically, simvastatin, penetrating the lipid bilayer, was able to induce an increased clustering of membrane microdomains, affecting cell response to pore-forming toxins. This lipid reorganization in the case of PLY treatment was characterized by an increase in cell survival related to a decrease in the membrane-binding ability of the toxin, as immunofluorescence and Western blotting data seem to suggest. We cannot exclude other additional pleiotropic effects of simvastatin behind the observed increase in cell viability, as already reported in a previous manuscript where the statin-conferred enhanced cellular resistance against PLY was calcium dependent [59]. However, in this study as well in other cases, the length of treatment with statins (≥24 h), makes it difficult to distinguish between classic cholesterol-lowering effects and pleiotropic ones. In our model, cells were pretreated with statins only for 1 h before the challenge with toxins and, in both cases, the total length of treatment was less than 24 h. Therefore, our observations are strictly related to the pleiotropic effects of statins, a topic accumulating enormous interest in recent years for their potential applications in different fields including cancer therapy and the treatment of Alzheimer’s and Parkinson’s diseases [60,61]. For example, a statin’s ability to change membrane bilayer properties could be beneficial for increasing sensitivity to cytotoxic drugs and defeating the multidrug resistance of tumor cells, while the observed pleiotropic effects in neuronal and glial cells could be beneficial for the treatment of neurodegenerative diseases [62].

The emerging evidence that statins and other pharmacological compounds can effectively modulate membrane bilayer properties, led in the last years to the development of a new field, named membrane-lipid therapy aimed at the identification and optimization of drugs capable of altering membrane lipid structures for pharmaceutical applications [63,64].

## Data Availability

Not applicable.

## Supplementary Materials

The following supporting information can be downloaded at: https://www.mdpi.com/article/10.3390/toxins14050346/s1, Figure S1: kinetic of MEL and PLY-treated HepG2 cell survival using MTT assay; Figure S2: Additional images showing that simvastatin-treatment increases rafts clustering.

## Abbreviations

CDCs	Cholesterol-dependent cytolysins
CTB	Cholera toxin B
DAPI	4′,6-diamidine-2-phenylindole
DMEM	Dulbecco modified Eagle medium
FBS	Fetal bovine serum
HD50	Hemolytic dose 50
LDH	Lactate dehydrogenase
MEL	Melittin
mβCD	methyl-β-cyclodextrin
MTT	3-(4,5-dimethylthiazol-2-yl)-2,5-diphenyltetrazolium bromide
NADH	Nicotinamide adenine dinucleotide reduced form
OD	Optical density
PBS	Phosphate buffer saline
PLY	Pneumolysin
RBC	Red blood cells
