# Supplementary material for: Membrane Cholesterol Content and Lipid Organization Influence Melittin and Pneumolysin Pore-Forming Activity
Source: Toxins (Basel). 2022 May 16;14(5):346. doi: 10.3390/toxins14050346 (PMC9147762; doi:10.3390/toxins14050346)
Supplement: Supplementary file 1 [file toxins-14-00346-s001.zip › toxins-1659873-supplementary.pdf]

Supplementary Materials: Membrane Cholesterol Content and Lipid Organization Influence Melittin and Pneumolysin Pore-Forming Activity

Shamish Ganpule, Akshay Kumar Vijaya, Aleksandra Sukova and Giulio Preta *

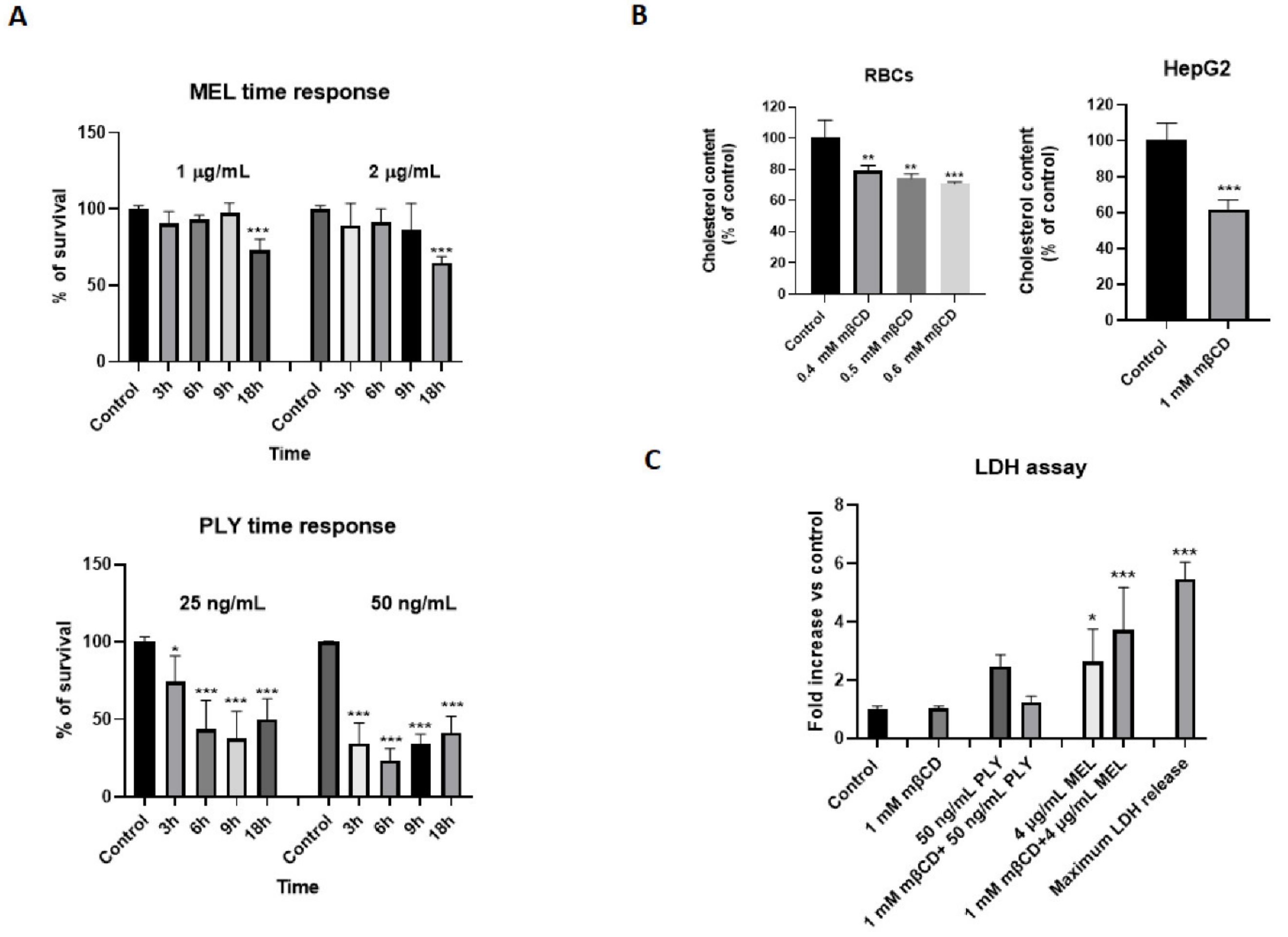

FigureS1 A: Kinetic of MEL and PLY-treated HepG2 cell survival using MTT assay. Cells were treated with the reported concentrations of the two toxins and for different time points. Data are presented as the arithmetic mean (SD) and statistical analysis was performed using one-way ANOVA Sidak's multiple comparisons test (* $p < 0.05$, *** $p < 0.001$). **B:** measurement of cholesterol levels in RBCs (left) and HepG2 cells (right) after treatment for 1h with the indicated concentrations of mβCD. Cholesterol levels were measured using the Amplex Red Cholesterol Assay kit and data are presented as % of cholesterol decrease compared to control (untreated) cells. Data are presented as the arithmetic mean (SD) and statistical analysis was performed using one-way ANOVA Sidak's multiple comparisons test for RBCs and an unpaired t-test in HepG2. **C:** LDH release measurement in HepG2 pre-treated with or without 1 mM mβCD and treated after 1h with MEL or PLY. Lactate dehydrogenase was quantified using CyQuant LDH Cytotoxicity assay and results are presented as fold increase compared to control. Maximum LDH release is a 10% lysis solution. Data are presented as the arithmetic mean (SD) and statistical analysis was performed using one-way ANOVA Sidak's multiple comparisons test (* $p < 0.05$, *** $p < 0.001$).

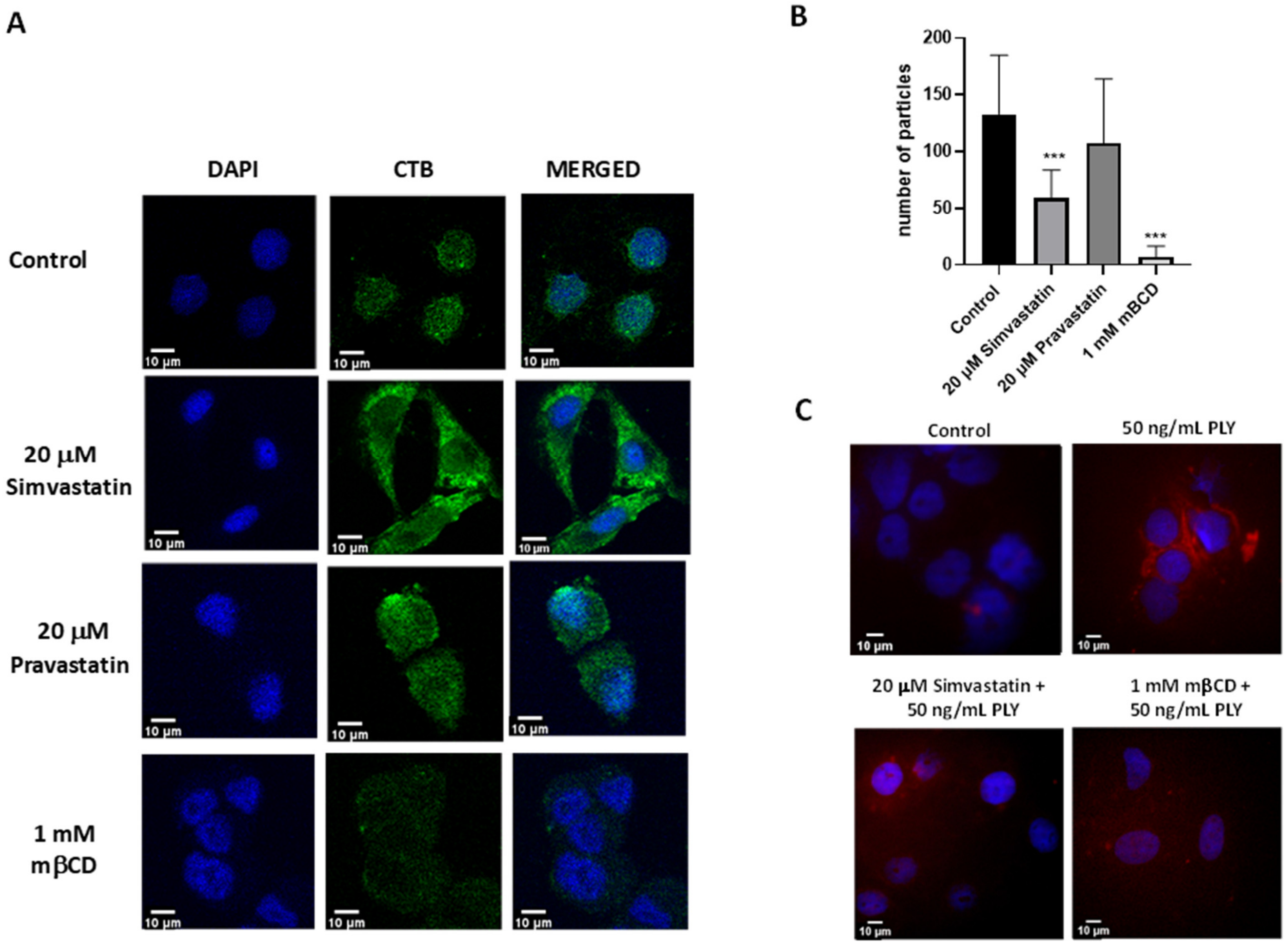

Figure S2. A: Additional images showing that simvastatin-treatment increases rafts clustering. Experiment performed as in figure 4A. Scale bar 10 µm. **B:** Simvastatin induces a decrease in the total number of particles. Pictures analysis was performed using Image J software and lipid rafts clustering was evaluated counting on the green channel, the total number of spots on at least 20 cells in different fields. Data are presented as the arithmetic mean (SD) and statistical analysis was performed using one-way ANOVA Sidak's multiple comparisons test (*** $p < 0.001$). **C:** Additional images showing that simvastatin-treatment reduces PLY binding to the membrane. Experiment performed as in figure 5A. Scale bar 10 µm.
